# The role of locomotion in psychological development

**DOI:** 10.3389/fpsyg.2013.00440

**Published:** 2013-07-23

**Authors:** David I. Anderson, Joseph J. Campos, David C. Witherington, Audun Dahl, Monica Rivera, Minxuan He, Ichiro Uchiyama, Marianne Barbu-Roth

**Affiliations:** ^1^Department of Kinesiology, San Francisco State UniversitySan Francisco, CA, USA; ^2^Department of Psychology, University of CaliforniaBerkeley, Berkeley, CA, USA; ^3^Department of Psychology, University of New MexicoNM, USA; ^4^Department of Physical Therapy, Samuel Merritt CollegeOakland, CA, USA; ^5^Department of Psychology, Doshisha UniversityKyoto, Japan; ^6^Laboratoire Psychologie de la Perception, Université Paris Descartes – Centre National de la Recherche ScientifiqueParis, France

**Keywords:** action, brain, cognition, crawling, locomotion, infancy, psychological development

## Abstract

The psychological revolution that follows the onset of independent locomotion in the latter half of the infant's first year provides one of the best illustrations of the intimate connection between action and psychological processes. In this paper, we document some of the dramatic changes in perception-action coupling, spatial cognition, memory, and social and emotional development that follow the acquisition of independent locomotion. We highlight the range of converging research operations that have been used to examine the relation between locomotor experience and psychological development, and we describe recent attempts to uncover the processes that underlie this relation. Finally, we address three important questions about the relation that have received scant attention in the research literature. These questions include: (1) What changes in the brain occur when infants acquire experience with locomotion? (2) What role does locomotion play in the maintenance of psychological function? (3) What implications do motor disabilities have for psychological development? Seeking the answers to these questions can provide rich insights into the relation between action and psychological processes and the general processes that underlie human development.

## Introduction

Locomotion is one of the most thoroughly studied behaviors in the animal kingdom. It has captivated the interest of engineers, ethologists, biologists, neurologists, clinicians, psychologists, and even philosophers. Most of the scientific interest in locomotion has centered on how it evolved, how it develops, how it is controlled, and how it can be rehabilitated following injury or disability. However, several theorists, from various epistemological traditions, have pondered whether locomotion makes a broader contribution to human life beyond its obvious role in moving from one place to another. For example, Mahler, a psychoanalyst, has stated that the onset of voluntary locomotion represents the “psychological birth” of the human infant (Mahler et al., 1975). Piaget (1952, 1954) argued that the origins of intelligence were in the intercoordination of sensory information with self-produced movements, including locomotion, and Gibson (1966, 1979) similarly stressed the importance of actions like locomotion for revealing meaningful information in the world.

Given the centrality of locomotion in such a diverse range of theoretical viewpoints, one might assume that the psychological correlates and consequences of the development of self-produced locomotion would be thoroughly understood. This is distinctly not the case. Only recently have the psychological consequences of self-produced locomotion been subjected to systematic empirical study (see Anderson et al., 2013 and Campos et al., 2000 for reviews). Researchers have shown that the onset of independent locomotion is indeed a pivotal event in the life of the human infant, heralding surprisingly broadscale changes in a variety of psychological functions, including perceptual-motor coordination, spatial cognition, memory, and social and emotional processes. Moreover, evidence reveals that locomotion is not merely a maturational antecedent to these psychological changes, but instead plays a causal role in their genesis (e.g., Uchiyama et al., 2008). Researchers have also begun to unravel the processes by which locomotion has its effects on psychological development, providing important insights into the mechanisms that underlie developmental change (e.g., Dahl et al., 2013).

The primary objective of the current paper is to describe a sample of the research linking locomotion to psychological development, highlighting the range of converging research operations—including variations of the classic *enrichment* and *deprivation* paradigms in animal studies—that have been used to isolate locomotion as a central contributor to these changes. A secondary objective is to highlight recent attempts to unravel the processes by which locomotion has its effect on psychological development. A final objective is to pose three questions to guide future research in this still relatively nascent, and often under appreciated, field of study. Before tackling these objectives, we will briefly address why empirical study of the psychological consequences of self-produced locomotion was neglected for so long. Placing the issue in historical context helps to show how the study of the psychological consequences of locomotor experience has challenged some of the core assumptions in developmental psychology. Pursuing the research agenda we outline in this paper can provide valuable insights not only into the processes that underlie developmental change but also into the broader linkage between action and psychological processes.

### Why have the psychological consequences of self-produced locomotion been neglected?

Although many theoretical traditions have highlighted the centrality of locomotion in human life, strong biases have existed in biology and psychology for much of the nineteenth and twentieth centuries against the notion that motoric activity plays a role in psychological processes or human development. Two factors have been particularly important in perpetuating this bias. First, a series of experiments in the 1930s failed to confirm that advanced motor development during infancy predicted advanced intellectual functioning later in life (Kopp, 1979), leading many psychologists to assume that motor activity was unimportant for psychological functioning. In hindsight, this line of research was ill conceived, posing questions that were too broad to be tested meaningfully and assuming that motor and intellectual development must be connected via a singular individual difference variable, like genetic integrity, that influenced both similarly. In addition, researchers failed to assess the domains of psychological function that were most likely to be affected by motor activity (ignoring the specificity principle, which states that each developmental change results from specific experiences in a specific context), and they also failed to consider that the role played by motor activity in psychological development might be easier to ascertain during developmental transitions when large and rapid changes occur simultaneously in motor and psychological functioning (Bertenthal and Campos, 1990).

The second factor perpetuating a bias against a role for motor activity, and by extension locomotion, in psychological development has been the domination of unidirectional models in psychological science and biological development. The two models that dominated psychological science for much of the twentieth century were the stimulus-response model and the information processing model. Both assumed that behavior was simply the end product of a chain of events that started with the reception of stimulation from the environment and ended with some type of action. Moreover, behaviorists were not concerned with psychological processes. Though cognitive processing intervened in the information processing model, adherents to that model were far more interested in those cognitive processes than the *less interesting* behavioral output and they didn't consider that action might reciprocally influence cognition and perception. In short, action was not considered relevant to the ontology of cognition—it was merely the output of processes that make use of cognition (Allen and Bickhard, 2013)—and whether the information for perception was self-generated or externally generated was irrelevant.

Similarly, in biology, the dominant model during most of the nineteenth and twentieth centuries was a nativist one that stressed the linear unfolding of a genetic blueprint. Genetic activity led to structural maturation, which in turn led to function, activity, and experience (Gottlieb, 2007). Again, adherents to this model did not consider that the relations between these different levels of analysis might be bi-directional. Even the empiricists (psychologists in this case), who trumpeted the importance of experience in human development, viewed development in linear terms, assuming that the environment exerted its effect on an essentially passive organism.

Nativism continues to hold sway amongst contemporary developmentalists (e.g., Spelke and Newport, 1998; Spelke and Kinzler, 2009), further perpetuating the bias against locomotion playing much of a role in psychological development. The preoccupation with documenting the origins of psychological phenomenon has led to confusion between what have been labeled *partial accomplishments* (Haith and Benson, 1998; Campos et al., 2000), the precursors to mature skills, and the mature skills themselves. The confusion in turn has minimized the importance of experience, particularly self-generated experience, in orchestrating qualitative reorganizations in behavior during postnatal development and short-circuited the analysis of the processes by which the substrates of skilled behavior, i.e., the partial accomplishments, are elaborated, differentiated, and inter-coordinated into full-blown skills (Campos et al., 2008; Kagan, 2008; Spencer et al., 2009).

### Why has the bias against locomotion begun to change?

The emergence and spread of bidirectional models in biology and psychology during the latter half of the twentieth century have led to greater acceptance of the idea that actions like locomotion might have consequences for psychological development. For example, dynamical systems theory and its close cousin ecological psychology stress the reciprocity between perception, action, and cognition, and view development as the result of a complex, contingent, and multi-determined web of interactions that emerge over time (Gibson, 1988; Thelen and Smith, 1994; Witherington, 2007, 2011). Similarly, Gottlieb's (e.g., 1970, 1991, 2007) notion of probabilistic epigenesis has provided a strong challenge to the unidirectional model of human development by highlighting the diversity of co-actions (reciprocal interactions that can literally change the interacting elements) that occur across the genetic, structural, and functional (environmental) levels of analysis during pre- and post-natal development. Probabilistic epigenesis states that development is a function of time-based, probabilistic relations between these different levels of analysis. Bidirectional models highlight the activity-dependent nature of structural and functional development and give experience an essential role in the developmental process.

Two aspects of probabilistic epigenesis are especially important to the empirical work linking self-produced locomotion to psychological development. The first is the idea that one developmental acquisition, like crawling, can generate experiences that bring about a host of new developmental changes in the same and different domains. These changes in turn create still other developments in a cascading cycle throughout the lifespan. From this perspective, individuals contribute to their own development by creating the experiences that drive developmental change. The second important aspect is the notion that experience does not have a singular effect on development; it can *induce* changes that are completely dependent on those experiences for their emergence, it can *facilitate* changes that would take place without such experiences, only more slowly, and it can *maintain* changes that have already taken place. Development is probabilistic because there is typically more than one ontogenetic pathway—although one of the many pathways (e.g., locomotor experience) may be the ordinary and expectable one. This line of thinking is clearly antithetical to the traditional unidirectional account of development in which developmental change is seen simply as the maturational unfolding of a genetic blueprint.

### What is special about locomotor experience?

Throughout the first year of life, infants gain control over an increasingly broader range of motor skills in a predictable sequence. Each new skill presents new opportunities to engage the world and exert a degree of control over it. What makes the acquisition of crawling—typically the first locomotor skill—so impactful is that it so dramatically changes the relation between the infant and her environment. No longer at the mercy of others for movement from one place to another, the infant now has an explosion of new goals to choose from and problems to solve. She can explore the environment and operate on it at will (Gibson, 1988). Exploration, in turn, provides new perspectives and it reveals new information and creates many novel experiences that can drive changes in a family of different psychological phenomena.

The breadth of these phenomena stems from the breadth of experiences that accompany locomotion. Moreover, these experiences do not simply represent “more of the same” because the experiences of the crawling infant are fundamentally different from those of the pre-crawling infant. Locomotion orchestrates this diversity of changes by making it almost inevitable that infants will encounter the experiences that contribute to specific psychological changes. The acquisition of independent locomotion is not only significant because of the breadth of psychological phenomena to which it is connected. Its enduring significance stems from the fact that once locomotion has been acquired it is available across the lifespan and so it may well be vital to the maintenance of the very psychological skills it had a role in bringing about. We will return to this point after first considering the role that locomotor experience plays in the ontogeny of two important phenomena: wariness of heights and the search for hidden objects.

## Locomotor experience and the emergence of wariness of heights

Wariness of heights is extraordinarily biologically adaptive, functioning to avoid falls that can maim, kill, and prevent reproduction of a person's genes. Indeed, Bowlby (1973) classified the fear of heights as one of the most salient “natural clues to danger.” Similarly, Gibson and Walk (1960) concluded that avoidance of dropoffs is evident in non-human animals and human infants at the first testing opportunity. Scarr and Salapatek (1970) described it as one of the two strongest fears observed in infants. It remains powerful even into adulthood, as is evident in the reactions of visitors to the transparent platform extending over the edge of the Grand Canyon (“The Grand Canyon's skywalk,” 2007), the Sears Tower, or a Shanghai skyscraper. It is no wonder that wariness of heights is considered under strong maturational control (Gleitman et al., 2007).

However, wariness of heights presents an enigma; it is not under maturational control, nor is it present at the earliest testing opportunity or when the threat of falling first materializes. Experience with locomotion seems to be a powerful factor in the onset of wariness of heights. Mothers notice two interesting phenomena related to dropoffs. First, there is a period after the onset of crawling when their infants would plunge over the edge of a bed, off the top of a changing table, or even off the top of a staircase if she were not extremely vigilant. Second, within 2–4 weeks of crawling onset, infants will avoid dropoffs. These maternal reports are highly consistent (Campos et al., 1978).

Laboratory experiments using a visual cliff confirm maternal reports. The visual cliff is a large table with a Plexiglas surface. Illuminated tiles immediately beneath the Plexiglas surface on the *shallow* side of the cliff give the impression of a solid surface, whereas the tiles four feet below the surface on the *deep* side give the compelling impression of a drop-off. Negative reactions to heights can be assessed by a number of indices of wariness, and each of these has been shown to undergo a developmental shift following the onset of locomotion. These indices include (1) changes from cardiac deceleration to acceleration when the infant is lowered to the deep side of the cliff (Campos et al., 1992); (2) initial crossing to the mother on a beeline when she calls the child over the deep side, followed by eventual avoidance (Campos et al., 1978); (3) initial absence of facial patterns indicative of distress when infants are lowered to the deep side of the cliff, to significant negative facial responses starting at 11 months of age and possibly before (Hiatt et al., 1979); and finally, a change from nonchalance to stiffening of the body and resistance with the arms when an infant is pushed from behind onto the deep side of the cliff. There is thus no doubt that a developmental shift takes place in wariness of heights. The shift is seen in many emotional ways and it is observed in real-world and laboratory contexts.

This developmental shift is where the enigma rests: by what process does the infant become wary of heights and how does that process produce a lifelong, biologically adaptive, wariness?

We can rule out the development of depth perception as the crucial factor. Infant depth perception is very well-developed some 2 or 3 months before wariness of heights is expectable (Timney, 1988). Depth perception is sufficiently well-developed at 6 months to allow clear differentiation of distances on the visual cliff. For instance, in a study by Walters (1980), prelocomotor 6-month-olds, when lowered toward the shallow or the deep side of the cliff, and who otherwise show no wariness of heights, extend their arms and hands in preparation for contact with the visually solid shallow side of the cliff, but show no such extension of arms and hands when lowered to the deep side. They quite happily land on their bellies on the deep side.

Falling experiences can also be ruled out as the crucial factor in the shift. The relation between falls and avoidance of heights or risky slopes is weak or non-existent (Walk, 1966; Campos et al., 1978; Adolph, 1997). Social referencing (Sorce et al., 1985) is not likely to play a role in the developmental shift either because it comes online well after the development of wariness of heights. So, the mother's facial, vocal, and gestural expressions cannot serve as unconditioned stimuli that become the basis for the infant learning to fear heights when paired with depth-at-an-edge (Mumme et al., 1996).

Finally, the developmental shift cannot be an artifact of the visual cliff apparatus. The solid glass surface cannot be said to provide a “safe” medium onto which the newly-locomoting infant can descend simply because touching the surface reveals its solidity. Though solid to touch, the transparent surface eventually becomes a source of avoidance with age and experience in longitudinally-tested infants (Campos et al., 1992). Furthermore, the maternal reports on infant near-falls cited above concur with the findings on the cliff, demonstrating ecological validity of findings using the cliff table. Lastly, there are the observations by Adolph (1997) using “risky slopes,” without a glass surface, that showed the same functional relation between locomotor experience and avoidance of dropoffs as does work with the visual cliff. The developmental shift found in visual cliff studies is thus robust, replicable, and ecologically valid.

### A proposed explanation of the ontogeny of wariness of heights

The explanation of the developmental shift toward wariness of heights must involve experience but not classical conditioning (such as to falls); it must involve the discovery of a factor or factors that provide an “affective sting” (i.e., concern relevance, Frijda, 1986) that the experience of depth alone does not provide; it must explain why the fear of heights is often accompanied by the reports of heights being “dizzying;” it must account for the role of locomotor experience in the shift; and it must explain the presence of wariness of heights in the occasional, though rare, prelocomotor infant. What can that factor or set of factors be?

Bertenthal and Campos (1990) proposed an explanation that meets the above criteria. They maintained that visual proprioception plays a crucial role in the onset and maintenance of wariness of heights. Although not widely known, visual proprioception is as fundamental a perceptual process as form, motion, depth, and orientation. Visual proprioception is the optically induced sense of self-movement produced by patterns of optic flow in the environment (Gibson, 1966, 1979). It is best known to most people by the experience, when one is seated stationary on a train or bus, of one's self moving when it is the train or bus on an adjacent track in the visual periphery that is moving. However, visual proprioception is much more than the source of a trivial illusion. It is crucial for establishing and maintaining postural stability and for navigation in the world. It is the apparent *loss of postural stability* linked to visual proprioception that leads to wariness of heights. According to Bertenthal and Campos, visual proprioception is not fully present in the infant with no locomotor experience, but becomes functional, and eventually well-established, as experience with locomotion increases. In brief, because of developmental changes in visual proprioception with locomotion, heights are initially not “dizzying,” but then become so.

**Figure 1:**
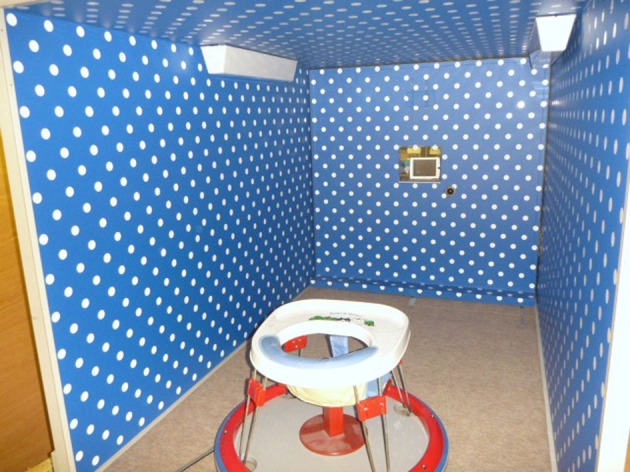
**The moving room.** Responsiveness to peripheral optic flow is determined by cross-correlating the infant's postural sway in the fore-aft direction, measured by four force transducers under the legs of the infant seat, with the movement of the side walls.

Visual proprioception depends on patterns of optic flow that covary with self-movement. When one is looking and moving straight ahead there is a radial (star-like) pattern with optical flow originating from a static point in the center of one's visual field. Simultaneously, there is a lamellar (layered and parallel) pattern of flow in the visual periphery. Although perception of self-movement has traditionally been relegated to information from the vestibular and the somatosensory systems, visual proprioception is so powerful that a standing 13-month-old infant will fall down when exposed to optic flow in a *moving room* (Lee and Aronson, 1974). The moving room is a small, textured enclosure with one end open (Figure 1). Pushing or pulling the room gives the child the perception of moving forward or backward (depending on the direction of optic flow) even when he or she is stationary. Peripheral lamellar optic flow, generated by moving only the side walls in the moving room, creates a particularly compelling sense of self motion and leads to greater visual-postural coupling than radial optic flow (Stoffregen, 1985). Visual proprioception is without doubt a powerful source of information for postural stability and instability.

Bertenthal and Campos (1990) linked visual proprioception to wariness of heights via the following set of propositions. First, they predicted that infants with locomotor experience would show visual proprioception in response to peripheral optic flow, whereas infants without locomotor experience would not, or would do so minimally. Secondly, once this type of visual proprioception comes online, it works in concert with vestibular, and somatosensory information to specify stasis or changes in posture or self-movement. Third, when a child approaches a dropoff, there is a sudden loss of visual proprioceptive information in the periphery, but not of vestibular or somatosensory information. At a dropoff, there is little or no optic flow in the periphery of the visual field and head/body movements produce little change in radial or lamellar flow because of the distance from the child to the closest visible surface (the floor). This loss of visual information is the basis for wariness of heights because of the disparity between visual and somatosensory/vestibular information for self-movement and/or a reduction in postural stability (see Brandt et al., 1980).

Locomotor experience is important in the functionalization of peripheral lamellar optic flow into visual proprioception for at least two reasons. One, the infant who is able to move voluntarily can notice and detect patterns of optic flow that coincide with forward and backward movements of the body. Prior to voluntary locomotion, there is little or no regularity between direction of optic flow and self-movement because when infants are carried passively, forward movement can be linked to any number of directions of optic flow depending on how the infants are held and where they are looking. In addition, most infants when carried early in life are in a state of “visual idle,” looking at nothing in particular. Only when the infant moves voluntarily do the head and eyes consistently point straight ahead (Higgins et al., 1996), allowing consistent exposure to radial optic flow in the central field of view and lamellar optic flow in the periphery. The second reason locomotor experience is important is that when the infant must navigate the world, it is important to segregate information about environmental features (specified in the central field of view) from information about self-movement (specified by peripheral optic flow) so as to steer an appropriate course and maintain postural stability (Gibson, 1979). Because these tasks must be accomplished simultaneously, locomotion leads to a perceptual differentiation wherein central and peripheral optic flow are relegated different perception-action functions. Attending to features of the environment can be accomplished more effectively and efficiently in the central field of view if postural stability is relegated to the periphery.

**Figure 2:**
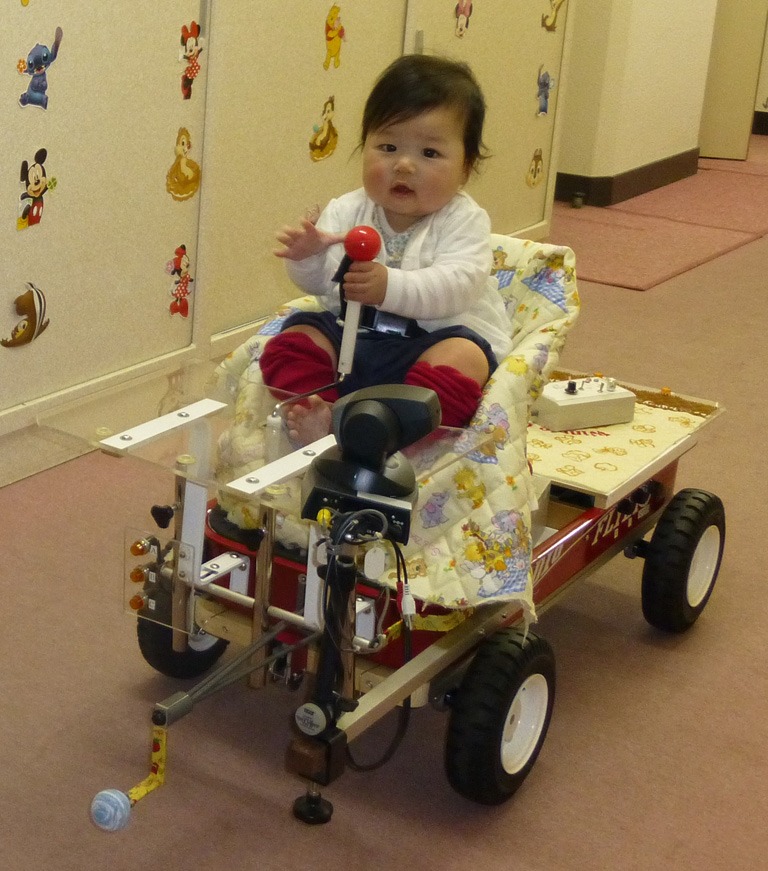
**The powered-mobility-device (PMD) used to test the relation between self-produced locomotion and psychological development.** Infants can move forward in the PMD by pulling on the brightly colored joystick.

There is now no doubt that locomotor experience affects visual proprioception. Using two converging research operations—(1) an age-held-constant study of locomotor, prelocomotor, and prelocomotor infants with artificial “walker” experience, and (2) the random assignment of precrawling infants to a condition in which they could control their own movement in a powered mobility device (PMD) (Figure 2) or a no-movement condition, Uchiyama et al. (2008) documented that infants with any kind of locomotor experience showed not only postural compensation to peripheral optic flow in a moving room, but also negative emotional reactions to peripheral optic flow, consistent with a sense of loss of postural stability. These findings confirmed previous reports of greater responsiveness to peripheral optic flow in infants with locomotor experience compared to same-aged infants without locomotor experience (Higgins et al., 1996). In sum, the proposition of the Bertenthal and Campos hypothesis that locomotor experience brings on or greatly improves visual proprioception has been empirically supported.

### Testing the link between visual proprioception and wariness of heights

Two studies were recently conducted by Dahl et al. (2013) to test the relation between visual proprioception and wariness of heights proposed by Bertenthal and Campos (1990). The first study examined whether newly crawling infants who were highly responsive to peripheral optic flow would be more likely to avoid heights. Wariness of heights was assessed on a visual cliff and postural compensation to peripheral optic flow was assessed by moving the side walls in a moving room. Under the infant's seat in the moving room were force sensors that recorded postural sway in the fore and aft directions. Cross correlating the postural sway data with the displacement of the side walls provided an index of the strength of the coupling between vision and posture.

**Figure 3:**
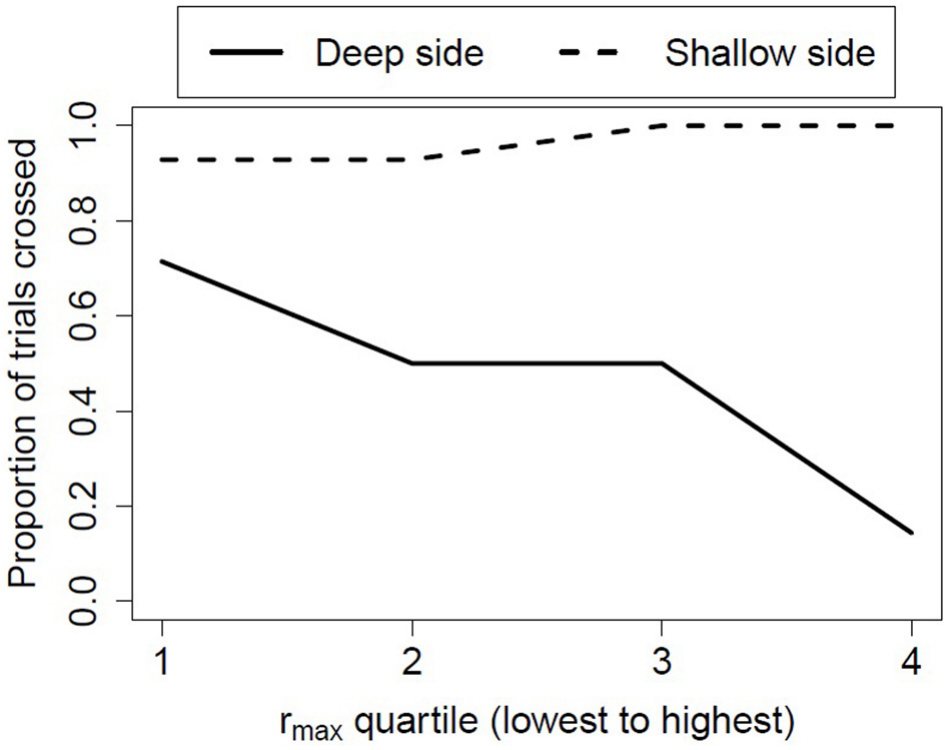
**The probability of crossing the deep or shallow sides of the visual cliff based on the infants' responsiveness to peripheral optic flow in the moving room**.

As predicted, postural compensation to peripheral optic flow was positively and significantly associated with infant avoidance of the deep side of the visual cliff. That is, the greater the coupling between an infant's postural sway and the wall movement, the more likely the infant was to avoid the drop-off. In contrast, there was no relation between visual-postural coupling in the moving room and avoidance of the shallow (non-drop-off) side of the visual cliff (see Figure 3). These findings were replicated in another unpublished study with somewhat younger infants who had similar amounts of locomotor experience, further evidencing the robustness of the relation between infant visual proprioception and wariness of heights.

The second study used the PMD to experimentally manipulate infant experience with self-produced locomotion and responsiveness to peripheral optic flow. The study had three purposes: (1) to investigate whether PMD experience would lead to increased wariness of heights, (2) to corroborate Uchiyama et al.'s (2008) finding that PMD experience leads to increased responsiveness to peripheral optic flow, and (3) to test whether the relation between PMD experience and wariness of heights is mediated by responsiveness to peripheral optic flow, as predicted by the Bertenthal and Campos (1990) hypothesis. Since all infants were precrawlers, they were tested on the visual cliff by measuring their heart rate (HR) while they were lowered onto the deep and shallow sides of the visual cliff. HR differentiation between the deep and shallow sides was used as an index of wariness (Ueno et al., 2012, showed that the crossing paradigm and the lowering paradigm on the visual cliff yield the same conclusions). As in the previous study, visual proprioception was assessed in the moving room.

**Figure 4:**
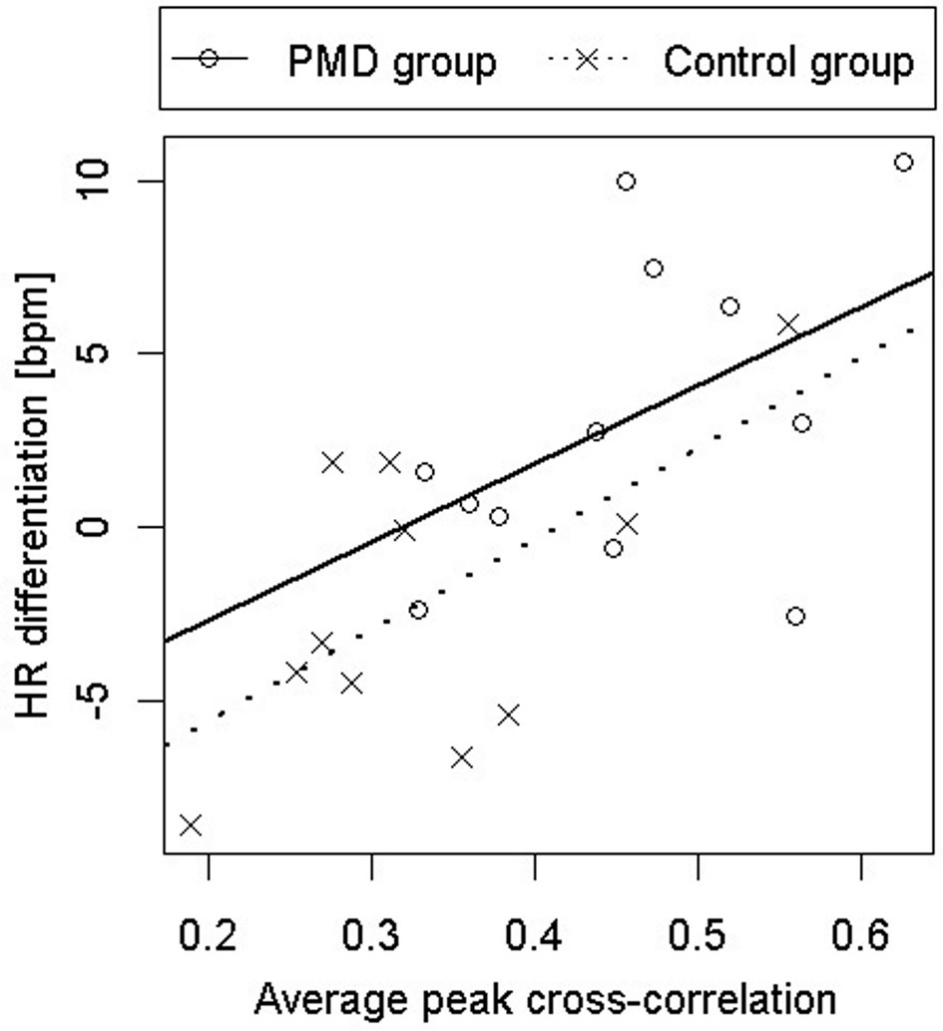
**Heart rate acceleration on the deep side of the visual cliff minus heart rate acceleration on the shallow side as a function of responsiveness to peripheral optic flow in infants who received powered-mobility-device (PMD) training and those who did not**.

**Figure 5:**
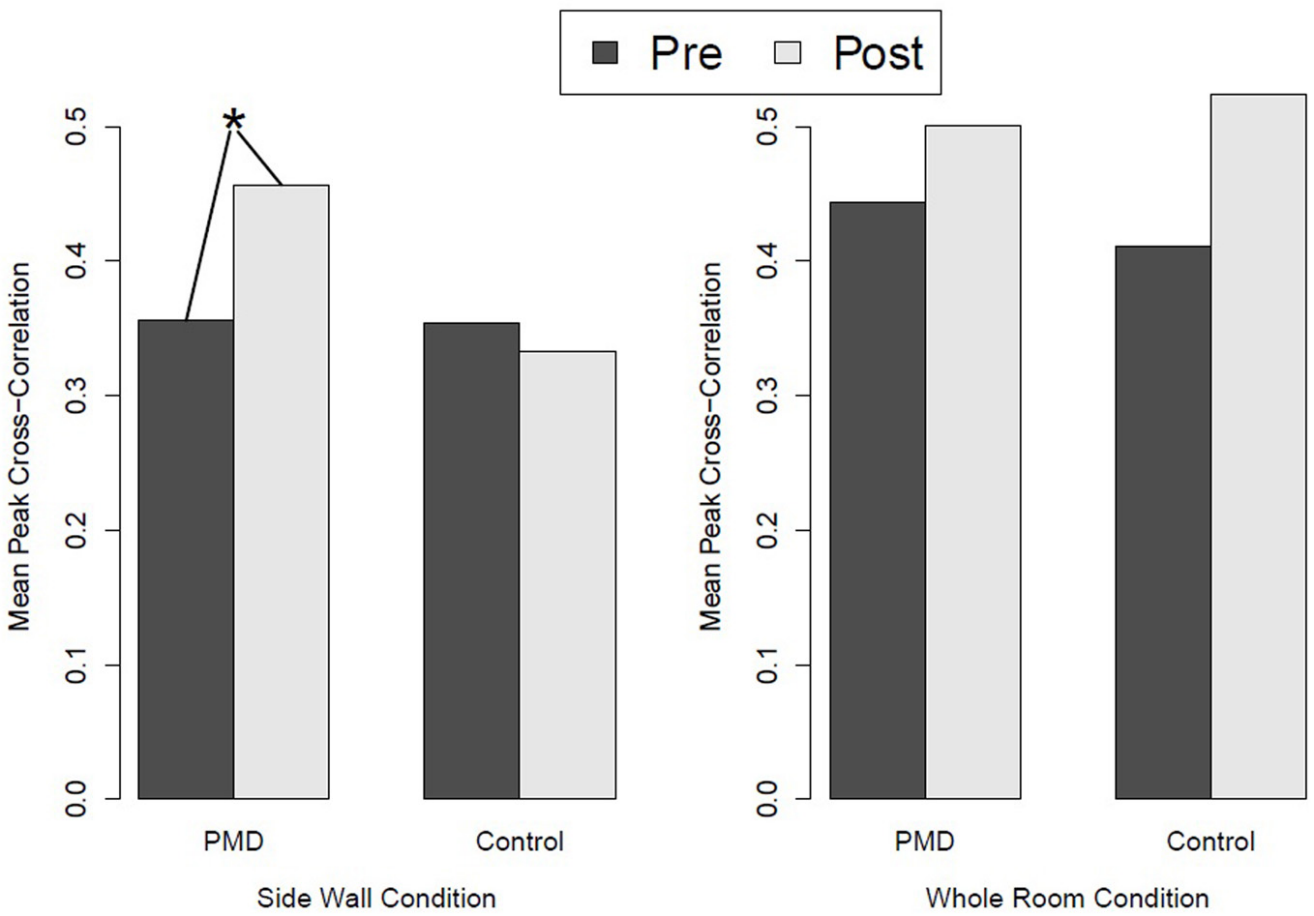
**Responsiveness to peripheral optic flow and global optic flow in the moving room in infants who received powered-mobility-device (PMD) training and those who did not.** ^*^p < 0.05.

All three predictions were supported. PMD infants showed greater HR differentiation between the deep and shallow sides of the visual cliff than control infants (see Figure 4), they showed greater responsiveness to peripheral optic flow in the moving room than controls (see Figure 5), and, finally, the relation between PMD experience and HR differentiation on the visual cliff was mediated by infant responsiveness to peripheral optic flow. In other words, only insofar as PMD infants had higher postural responsiveness to the moving room did they also show higher cardiac signs of wariness of heights.

The above studies thus show strong support for the hypothesis that wariness of heights typically comes about through locomotor-induced changes in visual proprioception. However, none of the studies actually manipulated infant use of visual proprioceptive information in the presence of a drop-off. The Bertenthal and Campos (1990) hypothesis implies that if crawling infants, ordinarily wary of drop-offs, are provided with additional visual proprioceptive information at the edge of a drop-off they should show less wariness of heights. The provision of visual referents has been shown to improve postural control at the edge of a drop-off in adults (Simenov and Hsiao, 2001).

In an ongoing study, a corridor was built on top of the visual cliff. The walls of the corridor are either covered by highly patterned fabric (increased texture condition) or are plain white (minimal texture condition). Importantly, the presence of the corridor gives no additional clues that the surface of the visual cliff is solid. Infants are encouraged by their mothers to cross the deep side of the visual cliff through the corridor. If infants rely on peripheral optic flow for postural stability as they locomote, and loss of that information leads to wariness when depth at an edge is encountered, then they should be more likely to cross the deep side of the visual cliff in the increased texture condition than in the minimal texture condition. Preliminary data conform to prediction. Infants with more than 6 weeks of crawling experience are significantly more likely to cross the deep side of the visual cliff in the increased texture condition than in the minimal texture condition. The added texture thus appears to provide optic flow that, at least in part, compensates for the loss of visual information at the edge of the drop-off.

In sum, convincing evidence has been provided for Bertenthal and Campos's novel explanation for the emergence of wariness of heights. Locomotor experience appears to functionalize peripheral optic flow such that infants come to rely on this source of visual proprioceptive information for postural stability during locomotion. Upon encountering a drop-off, infants show signs of wariness either because they lose information they have come to rely upon, they experience a discrepancy between information provided by the visual, vestibular, and somatosensory systems, and/or their postural stability decreases.

The above studies also show that locomotor experience is not the *only* way by which infants can become wary of drop-offs. Indeed, Dahl et al. (2013) reported a positive relation between responsiveness to peripheral optic flow and cardiac signs of wariness in the pre-locomotor control group. The development of wariness of heights, like so many other (if not all) developmental processes is not deterministic, but probabilistic (Campos et al., 2000; Gottlieb, 2007). Transitions typically engendered by locomotor experience, like reliance on peripheral optic flow for visual proprioception, can sometimes be brought about through alternative developmental pathways. One question for future research is what these additional developmental pathways are in the cases of visual proprioception and wariness of heights.

### Summary

Converging research operations—including the experimental manipulation of infant experience with self-produced locomotion—have systematically documented that locomotor experience can induce a reorganization in visual proprioception and the onset of wariness of heights. These same converging operations have begun to address issues of process by establishing functionalization of peripheral optic flow as an experiential mediator in the relation between self-produced locomotion and wariness of heights. As such, this line of research serves as a model for beginning to tackle the question of how locomotor experience might bring about its functional consequences for other psychological skills. In the next section, we examine the relation between locomotor experience and improved search for hidden objects. Though the link between the two is strong and the processes that underlie the link are extremely important to understand, it has not yet received the same rigorous experimental treatment as the link between locomotion and visual proprioception and wariness of heights.

## Locomotor experience and manual search for hidden objects

Correctly searching for an object hidden in one of two locations proves to be a surprisingly difficult skill for the infant who has already developed proficiency in reaching and grasping. Infants between 8 and 9 months-of-age can successfully retrieve an object hidden within reach at one location, but they often fail when the object is hidden under one of two adjacent locations, even when the locations are perceptually distinct (Piaget, 1954; Bremner, 1978). More curiously, infants at this age will often continue to search for an object in its original hiding location even after they have seen it moved to a new hiding location. This perseverative search is referred to as the *A-not-B error* and the infant's performance becomes progressively poorer as the delay between hiding in the new location and search increases (Diamond, 1990).

The ability to search for and retrieve hidden objects has been the subject of intense scientific scrutiny because it represents a major transition in the infant's understanding of spatial relations. The capacities that underlie successful spatial search are thought to contribute to many important cognitive changes, including concept formation, aspects of language acquisition, representation of absent entities, the development of attachment, and other emotional changes (Haith and Campos, 1977). Importantly, changes in spatial search behavior have been explained entirely in maturational terms; specifically, maturation of the dorsolateral prefrontal cortex has been postulated as the necessary precursor to successful search (Kagan et al., 1978; Diamond, 1990). In contrast, Piaget (1954), among others (e.g., Hebb, 1949), has argued that changes in search behavior stem from motoric experience and active exploration of the world.

### Evidence linking locomotion to skill in spatial search

A number of researchers, including Piaget (1954), have speculated about a link between skill in spatial search and locomotor experience (Bremner and Bryant, 1977; Campos et al., 1978; Acredolo, 1978, 1985; Bremner, 1985). The first confirmation of the link was provided by Horobin and Acredolo (1986) who showed that infants with more locomotor experience were more likely to search successfully at the B location on a series of progressively challenging hiding tasks. The finding was replicated and extended by Kermoian and Campos (1988), using a similarly challenging series of spatial search tasks that ranged from retrieving an object partially hidden under a single location to the A-not-B task with a seven-second delay between hiding and search. Infants in the study were all 8.5 months-of-age but differed in experience with independent locomotion. The results showed clearly that infants with hands-and-knees crawling experience or experience moving in a wheeled-walker significantly outperformed the prelocomotor infants on the spatial search tasks. Moreover, search performance improved as experience with locomotion increased. For example, 76% of crawling and walker infants with nine or more weeks of locomotor experience successfully searched in the B location on the A-not-B test with a 3 s delay compared to only 13% of infants without locomotor experience.

The obvious conclusion from the Kermoian and Campos (1988) study is that locomotion, regardless of how it is accomplished, makes an important contribution to spatial search. However, a third experiment in the series raised an important caveat to that conclusion. *Belly crawling* infants, who were the same age as those tested in experiments 1 and 2, with between 1 and 9 weeks of crawling experience performed like *prelocomotor* infants on the spatial search tasks. Moreover, no relation was found between the amount of belly crawling experience and spatial search performance.

Why would belly crawling experience *fail* to make the same contribution to skill in spatial search as hands-and-knees crawling and walker experience? Kermoian and Campos (1988) argued that belly crawlers failed to profit from their locomotor experiences because belly crawling is so effortful and inefficient. Belly crawlers were thought to devote so much effort and attention to organizing forward progression that they were unable to deploy attention to the environment in the same way as the hands-and-knees crawlers and infants in walkers. Consequently, the belly crawlers may not have noticed some of the important spatial transformations during crawling, such as occlusion and reappearance of objects that contribute to improved search performance.

The Kermoian and Campos (1988) findings have been replicated and extended using a variety of converging research operations, including cross-sectional and longitudinal research designs as well as a variation of the deprivation design that took advantage of ecologically and culturally mediated delays in the onset of independent mobility in urban Chinese infants (Tao and Dong, 1997, unpublished data). Specifically, infants in Beijing who were delayed in locomotion by 2 to 4 months relative to North American norms initially performed poorly on the A-not-B test, then improved dramatically as a function of locomotor experience regardless of the age at which they acquired independent locomotion.

The relation between locomotor experience and spatial search performance is not confined to typically-developing infants. The relation has also been confirmed in a longitudinal study of seven infants with spina bifida (Campos et al., 2009). Spina bifida is a neural tube defect that is associated with delays in locomotor and psychological development. The test was a two-position hiding task in which a toy was hidden only in one location, with a second hiding location serving as a distractor. Infants were tested monthly after recruitment until 2 months after the delayed onset of independent locomotion, which occurred at 8.5, 11.5, and 13.5 months-of-age in three of the infants and 10.5 months-of-age in the other four. Dramatic improvements on the task were noted following the onset of locomotion. Infants searched successfully for the hidden object on only 14% of trials before they were able to crawl, but improved to 64% correct search following the delayed onset of locomotion.

**Figure 6:**
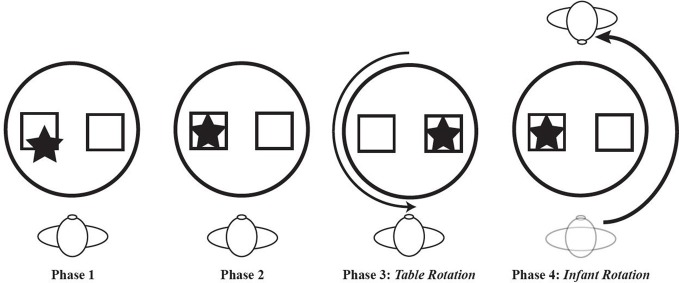
**Four phases of a hypothetical spatial search task.** In phase 1, the object is partially hidden by an occluder. In phase 2, the object is completely hidden by the occluder. In phase 3, the object is completely hidden on the left side but the table is rotated 180 deg before the infant is allowed to search. In phase 4, the object is hidden and the infant is rotated 180 before search is permitted.

Bai and Bertenthal (1992) studied the link between locomotor experience and spatial search in the context of a paradigm designed to assess position constancy. Position constancy is an ability to find an object or location following a shift in one's spatial relation to that object or location. Position constancy would be impossible without a basic level of skill in spatial search. Three groups of 33-week-old infants were tested. One group was prelocomotor, one group had 2.7 weeks of belly crawling experience, and one group had 7.2 weeks of hands-and-knees crawling experience. An object was hidden under one of two different colored cups that were placed side by side in front of the infant. Prior to searching for the object, the infant was rotated 180 deg around the other side of the table on which the cups were placed *or* the table was rotated 180 deg. The data from the first trial showed a particularly strong effect of locomotor experience. Infants with hands-and-knees crawling experience successfully retrieved the object on 72% of trials following rotation to the other side of the table compared to a 25% success rate for the prelocomotors. As in Kermoian and Campos's (1988) spatial search experiment, the belly crawlers in Bai and Bertenthal's study performed liked prelocomotors, searching successfully on only 30% of trials. Notably, the groups did not differ on their search performance when the table was rotated, likely because this type of displacement is rarely experienced by any infant, regardless of locomotor experience. (Figure 6 shows a hypothetical series of spatial search tasks to highlight the difference between the typical search procedure and the one in which the table or the infant is rotated).

### How is spatial search facilitated by locomotor experience?

The process by which locomotion contributes to spatial search remains poorly understood despite the range of converging research operations that have been used to document the link between locomotor experience and skill at spatial search. The need to explain the spatial component of manual search for hidden objects (where is the object located) as well as the temporal component (improved tolerance of increasing delays between hiding and search) has added to the challenge of developing viable explanations. Nevertheless, we have speculated previously (Campos et al., 2000) that at least four different factors contribute to improvements in search performance: (1) shifts from egocentric to allocentric coding strategies, (2) new attentional strategies and improved discrimination of task-relevant information, (3) improvements in means-ends behaviors and greater tolerance of delays in goal attainment, and (4) refined understanding of others' intentions.

#### A shift in coding strategies

Piaget first proposed that changes in spatial search performance reflect shifts from egocentric (body referenced) to allocentric (environment referenced) coding strategies (Piaget, 1954). He reasoned that prelocomotor infants could rely on egocentric coding strategies because they interacted with their environment from a stationary position. Thus, an object on the left would always be found on the left and an object on the right would always be found on the right. However, egocentric coding strategies are unreliable once the infant starts to move from place to place because the mobile infant's relation to the environment changes constantly. In Piaget's scheme, objects are first tied to the sensory impressions they give rise to and then to the actions that are performed on them. Even when infants can first represent objects independently of their own actions, the objects are still bound to specific locations in space. Only after infants develop a truly objective view of the world do they realize that objects can potentially inhabit many different positions in space.

#### New visual attentional strategies

Locomotor infants are commonly observed to be more attentive and less distractible during spatial search tasks (Campos et al., 2000). The idea that locomotion might facilitate changes in attentional strategies is quite reasonable if one assumes that attention is largely in the service of actions (e.g., Franz, 2012). Richard Walk has been one of the most vocal proponents of this idea, arguing that, “Although motor activity is important, its function seems to be mainly that of properly directing attention; the motor activity itself seems to contribute little” (Walk, 1981, p. 191).

Acredolo and colleagues first proposed visual attention as a mediator between locomotor experience and success on spatial search tasks (Acredolo et al., 1984; Acredolo, 1985; Horobin and Acredolo, 1986). They noticed that infants who kept an eye on the hiding location were more likely to retrieve the object successfully. In addition, visual distractions that encourage the infant to take their eye off the hiding location decrease the likelihood of successful search (Diamond et al., 1994). Keeping an eye on objects may be a particularly helpful way for a locomotor infant to retrieve objects following self-displacement. Keeping an eye on objects may also help infants to discriminate perceptually relevant information about the self and the environment through the process of *education of attention* to meaningful invariants (Gibson, 1979). Improved spatial discrimination of relevant task features has been proposed as one means by which locomotor experience might facilitate performance on the A-not-B task (Smith et al., 1999; Thelen et al., 2001).

#### Improvements in means-ends behaviors and working memory

Improvements in means-ends behaviors (e.g., Diamond, 1991) and greater tolerance for delays between initiating a behavior and completing it have been proposed to account for the observation that errors on the A-not-B task increase as the delay between hiding and search increases. How is experience with locomotion implicated in this process? The logic is that prone locomotion is a continuous task that is accomplished by concatenating a series of discrete movements of the arms and legs. The infant often struggles with several different means of coordinating all four limbs before discovering the diagonal pattern of couplings between the arms and legs that characterizes proficient (and efficient) four-limbed gait (Freedland and Bertenthal, 1994; Adolph et al., 1998). Learning to locomote proficiently may then transfer to learning other means-ends behaviors, perhaps through a process akin to *learning how to learn* (Harlow, 1949; Adolph, 2005; Seidler, 2010). In addition, locomotor goals require more time to complete than discrete actions like reaching and so the infant must keep the locomotor goal in mind for a longer period of time, taxing working memory.

A recent study linking locomotor experience to greater flexibility in memory retrieval provides indirect evidence that locomotion might facilitate the infant's ability to tolerate longer delays in the A-not-B task. Herbert et al. (2007) tested 9-month-old crawlers and non-crawlers on a deferred imitation task. An experimenter demonstrated an action on a toy and the infants were tested 24 h later to see if they would perform the same action. Crawlers and pre-crawlers imitated the action when they were given the same toy in the same context in which they were tested (laboratory or home), however, crawlers were significantly more likely than pre-crawlers to imitate the action when the toy and the testing context were different. The authors argued that locomotor experience promotes flexibility in memory retrieval because locomotor infants have abundant opportunities to deploy their memories in novel situations. It is not unreasonable to think that locomotion might also contribute to changes in working memory given that it has been linked to long-term memory. Such changes would be the basis for the greater tolerance of delays in hide-and-seek tasks.

#### Improved understanding of others' intentions

We have already noted that locomotor infants are more attentive and less distractible during search tasks. However, they also appear to search for communicative signals from the experimenter. This search is likely related to their ability to follow the referential gestural communication of an experimenter (e.g., Campos et al., 2009) and increased distal communication with the parent after the onset of locomotion (Campos et al., 2000). The importance of social communication in the A not B error has recently been highlighted by an experiment showing that perseverative search errors are considerably reduced when communication between the experimenter and infant is minimized (Topál et al., 2008). The authors argue that infants make the error because they misinterpret the *game* they are playing with the experimenter during the trials when objects are hidden at the A location. The growing literature on the link between action production and action understanding (e.g., Sommerville and Woodward, 2010) is also relevant to the potential mediating role of understanding others' intentions in successful spatial search. This literature suggests that infants' understanding of other people's actions as being goal-directed is a function of their own action experience.

### Summary

The evidence supporting a link between locomotor experience and spatial search performance is compelling. A range of converging research operations have shown that infants who can locomote perform better on spatial search tasks than infants who cannot. However, it is important to note here that we have not yet demonstrated a *causal* association between locomotion and spatial search performance as has been done for locomotion and visual proprioception and wariness of heights. The PMD is currently being used to conduct the pivotal studies. In addition, more attention must be devoted to understanding *how* locomotor experience contributes to spatial search performance. While the proposed mechanisms described above seem intuitive and viable, none have been confirmed experimentally.

The need for better understanding of the developmental process prompts us to raise additional questions about the relation between locomotion and psychological development that have received scant attention in the research literature. These include, how does the brain change when infants acquire locomotor experience, what role does locomotion play in the maintenance of psychological function, and what implications do limitations in motor ability have for psychological development? We now turn our attention to these important questions in the hope of showing how they can contribute to a deeper understanding of the processes that link action and psychological function and the processes that underlie developmental change.

## What changes in the brain occur when infants acquire experience with locomotion?

The emergence in infancy of each new motor skill brings new means of engaging the world. Given the activity-dependent character of neurological development highlighted by contemporary, bidirectional developmental models, we should expect reorganizations in cortical structure to accompany and be dependent on the acquisition of these skills. Surprisingly little empirical work, however, exists to confirm this speculation. Thus, the question of what changes in the brain are consequences of acquiring independent locomotion remains largely unexplored.

The critical role that activity plays in the development of psychological function extends to the development of neurological structure and function. Empirically, the activity-dependent character of neurological development is now well-established (Katz and Shatz, 1996; Pallas, 2005; Gottlieb et al., 2006; Westermann et al., 2007). Consider the oft-cited example of ocular dominance column formation, in which binocularly innervated tissue in layer 4 of the visual cortex developmentally segregates into alternating, *eye-specific* columns of cortical neurons. Even brief monocular deprivation in early postnatal development—limiting sensory activity to one eye—produces major anatomical changes to the structure of these columns (Hubel and Wiesel, 1963; Katz and Crowley, 2002). Such functional restructuring of the cortex illustrates how its eye-specific layering is plastically responsive to activity-derived competition for cortical neuronal resources (Katz and Shatz, 1996; Mareschal et al., 2007), even in premature infants (Jandó et al., 2012).

At the more macro-level of organismic activity, numerous examples of activity-modified brain structure exist, from demonstrations of cortical reorganization when novel motor skills are learned (e.g., Karni et al., 1998; Kleim et al., 1998; Zatorre et al., 2012) to the classic environmental complexity studies of Rosenzweig and colleagues, which show structural changes in the brains of rats reared in complex environments and given opportunities to actively explore and play with various objects compared to rats that were visually exposed to the complex environment but unable to engage with it. Among the structural changes are increases in synaptic size and density, expanded dendritic arborization, and increases in glial cells, vascular density, and neurogenesis (e.g., Ferchmin et al., 1975; Greenough et al., 1987; Markham and Greenough, 2004; Vazquez-Sanroman et al., 2013).

The importance of micro and macro levels of activity for the development of neurological structure is not just limited to modifications or extensions of existing neural architectures. Even *in utero*, before sensory systems are functionally active and sampling external stimulation, sensory neurons engage in *spontaneous* waves of activity that influence cortical differentiation (O'Leary, 1989; Pallas, 2005; Mareschal et al., 2007). Alongside this spontaneous neural activity is internally generated spontaneous activity issuing from cortical and subcortical structures of the brain. Such activity is considered by many to serve a critical role in the formation and early differentiation of neural networks (O'Leary, 1989; Katz and Shatz, 1996; Westermann et al., 2007). For example, the emergence of initial column structure in layer 4 of the visual cortex depends on spontaneously generated retinal activity (Feller and Scanziani, 2005; Mareschal et al., 2007) and experimental blockage of such activity has adverse consequences for neural development (Pallas, 2005). This also holds true at the macro level for the spontaneous motor activity of embryos and fetuses during prenatal development; experimental restraint of such activity yields morphological abnormalities in skeletal, muscular, and neural development (Einspieler et al., 2012).

In short, functional activity plays a central role in the formation, construction and development of structure in the nervous system. In stark contrast to the unidirectional framing of structure-function relations featured within traditional, maturational treatments of brain development, more and more neurologically-focused empirical work argues that function and structure reciprocally influence on one another throughout development. The bidirectionality of the relationship situates functional activity at the very heart of structural development, not as a mere epiphenomenal outgrowth of it. Such bidirectionality in structure-function relations is the hallmark of Gottlieb's (1970, 1991, 2007; Gottlieb et al., 2006) *probabilistic epigenesis* and is a mainstay of more recent efforts to establish relational approaches to neurological development, such as the theoretical framework of *neuroconstructivism* (Johnson and Karmiloff-Smith, 2004; Mareschal et al., 2007; Westermann et al., 2007).

What, then, do we know about the influence that locomotion has on the brain? The limited insights we have into the brain changes that accompany the onset of crawling come from work that was done by Bell and Fox (1996, 1997). They used an age-held-constant design with 8-month-olds who varied in their experience with hands-and-knees crawling activity to investigate the relation between cortical development and crawling experience. In their first study, four groups of infants—a prelocomotor group, a novice crawling group (1–4 weeks of experience), a middle-level crawling experience group (5–8 weeks of experience), and a long-term crawling experience group (9 or more weeks of experience)—were compared using a measure of EEG coherence across frontal, parietal, and occipital brain regions to index synaptic connectivity. EEG coherence measures the degree of association or coupling between different brain regions.

Bell and Fox (1996) discovered a curvilinear relationship between crawling experience and EEG coherence. Specifically, infants with 1–4 weeks of crawling experience demonstrated much greater EEG coherence than their long-term crawling counterparts (9 or more weeks of experience) and their prelocomotor counterparts. In their second study, Bell and Fox (1997) reproduced the same basic curvilinear relationship across the four groups of crawlers, however, this time with an estimate of within-region EEG power. The relationship held for EEG power in the frontal and parietal regions of the brain, but not the occipital region. Again, it was the infants with 1 to 4 weeks of crawling experience who demonstrated greater EEG power values than all other groups.

Given the greater coherence and power seen in the group with minimal crawling experience, Bell and Fox (1996, 1997) concluded that the brain changes represented an *experience-expectant* rather than an *experience-dependent process* (Greenough et al., 1987; Greenough and Black, 1992). As their labels suggest, experience-expectant processes are thought to emerge in anticipation of experiences that are ubiquitous and common to all members of a species, whereas experience-dependent processes are idiosyncratic or unique to an individual. Bell and Fox argued that the brain overproduced synaptic connections in anticipation of the new sets of experiences likely to derive from the acquisition of crawling, a species-typical motor skill. Synaptic pruning was assumed to follow the initial overproduction of synapses as the infant consolidated crawling and its experiential consequences.

Do the changes in EEG coherence and power seen at the onset of crawling really represent an experience-expectant rather than an experience-dependent process? Unfortunately, we don't have an answer to this question as no attempts have been made to replicate the Bell and Fox experiments. Two factors lead us to believe that the observed changes were dependent on experience, however. First, though the infants in the two studies had limited crawling experience, it must be remembered that they were hands-and-knees crawlers. This is important because infants typically explore many different forms of prone locomotion before converging on the more efficient hands-and-knees pattern, as noted earlier in the paper (Adolph et al., 1998). Consequently, Bell and Fox may have underestimated the amount of experience the infants had with self-generated locomotion. Second, an explosion of research in the neurosciences over the last decade has documented countless examples of experience-dependent plasticity in human development across the lifespan.

When the results from the environmental enrichment studies alluded to earlier are combined with the role that functional activity is known to play in the development of the nervous system, the idea that locomotion induces changes in the brain seems eminently reasonable. Nevertheless, the idea awaits experimental confirmation. Here is another research question that could be addressed using the powered-mobility-device. We hypothesize that prelocomotor infants given training in the PMD would show similar EEG coherence and power values to those seen in the infants with 1–4 weeks of crawling experience in the Bell and Fox (1996, 1997) studies and higher values than seen prior to training. In contrast, we would not expect to see changes in coherence and power in infants who did not receive training.

## What role does locomotion play in the maintenance of psychological function?

We noted earlier in the introduction that Gottlieb (1970, 1991, 2007) outlined three roles for experience in development—induction, facilitation, and maintenance. The discussion so far has focused on the first two roles; it is now time to focus on maintenance, the role that has received little, if any, empirical attention in the developmental literature. The concept of maintenance by experience has enormous implications for our understanding of the declines in psychological function associated with the aging process, and it provides a theoretical bridge between the processes that generate psychological structure and function in the early years of life and those that contribute to its deterioration later in life.

Experientially-induced cognitive and neural plasticity during adulthood is a topic of major interest in the neurosciences at the moment because of the dramatic shift in the proportion of the global population that will be over 65 years-of-age within the next 25 years and the concomitant personal, social, and economic costs that stem from age-related declines in cognitive function (Anderson-Hanley et al., 2012; Karbach and Schubert, 2013). It is particularly relevant to the central thesis of this paper that changes in an older person's gait are now recognized as early predictors of dementia, including Alzheimer's disease (Hall et al., 2000; Verghese et al., 2002, 2007). Those individuals at risk for dementia have slower walking speeds, disrupted rhythms, and show greater variability from stride to stride. Equally relevant is the prevailing tendency to view gait dysfunction as the first symptom of the disease rather than a contributor to the disease. In other words, most researchers assume that gait dysfunction (and motor dysfunction more broadly) is simply the earliest manifestations of the neural and vascular changes that will ultimately lead to detectable cognitive impairment, even though many acknowledge that the relation between physical activity and cognitive function is complex and likely reciprocal (Cedervall et al., 2012).

The tendency to downplay or ignore a potential role for mobility impairment in the progression of cognitive impairment is surprising given what is now known about the protective effects of physical activity on cognitive functioning in the elderly. (However, it is reminiscent of the skepticism that has met the idea that locomotion contributes to early psychological development.) Numerous studies have shown a positive effect of exercise and physical fitness on mental health and cognitive performance, using correlational research designs and randomized controlled trials (for reviews see Kramer and Erickson, 2007; Hillman et al., 2008; Baker et al., 2010; Chaddock et al., 2010; Erickson et al., 2012). Moreover, the areas of the brain where the most dramatic exercise-related structural changes occur, the neural, vascular, and molecular substrates that underlie these changes, and the effects that can be attributed to exercise *per se*, vs. learning, have been well-documented (Nithianantharajah and Hannan, 2009; Thomas et al., 2012).

The differential effects of learning vs. exercise on brain development, demonstrated some years ago by Greenough and colleagues (Black et al., 1990), and the brain regions known to be affected by physical activity, are important to consider relative to the potential effects of locomotion on the maintenance of psychological function. Rats who were given a prolonged period of wheel running showed an increase in blood vessel density in the cerebellum whereas those given acrobatic training showed an increase in synaptogenesis. More recent work has shown that while exercise can increase neurogenesis in the mouse hippocampus, environmental enrichment enhances the survival of new neurons and increases the likelihood they will be incorporated into existing neural networks (Kronenberg et al., 2003).

Exercise-related changes in the brain are typically localized to the motor cortex, the cerebellum, and the hippocampus (Thomas et al., 2012). Although the cerebellum has traditionally been assumed to participate exclusively in the control of movement, Diamond (2000) has argued that the connections between the cerebellum and the dorsolateral prefrontal cortex suggest that the cerebellum might also play an important role in cognitive functions. Deterioration in the hippocampus, which plays a central role in learning, memory, and spatial skills like navigation, precedes and leads to memory impairment, Alzheimer's disease, and depression in older adults (Thomas et al., 2012). A recent randomized controlled trial showed that a 12 month exercise program (walking) led to increases in the size of the anterior hippocampus and improved spatial memory in older adults (Erickson et al., 2011).

Having noted the different effects of exercise vs. environmental enrichment on the brain, one wonders whether the changes in hippocampal size noted by Erickson et al. (2011) were a function of the physiological demands of walking or the engagement with the environment that walking permits. A recent study on exergaming (a combination of exercise and video game play) sheds some light on this issue. Anderson-Hanley et al. (2012) randomly assigned older adults to a cybercycling intervention, which involved virtual reality tours through simulated environments and competition with other cyclists, or to a traditional cycling intervention on a stationary bike. Despite equivalent levels of effort and fitness, the cybercyclists showed significantly greater improvements in cognitive function following the intervention than traditional cyclists. Importantly, cybercyclists showed significantly larger increases in brain derived neurotrophic factor (BDNF), an important neurotrophin thought to mediate exercise-induced neurogenesis and synaptogenesis, than traditional cyclists. Thus, exercise with simultaneous cognitive engagement was a much more effective facilitator of cognitive function than exercise alone.

Finally, it is highly relevant to again note the role played by the hippocampus in spatial navigation to fully appreciate the potential impact that locomotion has on the maintenance of psychological function. Interactions with complex environments place highly specific demands on navigation and lead to measurable changes in the hippocampus. For example, London taxi drivers, who are held to some of the most rigorous standards in the world relative to knowing their city, have greater gray matter volume in the mid-posterior hippocampi. Moreover, greater driving experience is associated with greater posterior hippocampal gray matter volume (Maguire et al., 2000, 2006). Many complex navigational processes decline with hippocampal atrophy (Nedelska et al., 2012).

In an interesting parallel with the developmental work linking the onset of crawling to the increased use of allocentric spatial coding strategies (note, much of that work was not covered in the current paper, but see Anderson et al., 2013 for a recent review), researchers have shown that allocentric spatial coding strategies in healthy older adults correlate with gray matter volume in the hippocampus whereas egocentric strategies correlate with volume in the caudate nucleus (Konishi and Bohbot, 2013). A study by Harris et al. (2012) has recently shown that aging specifically impairs the ability to switch from an egocentric to an allocentric navigational strategy during a virtual maze task. This finding is important to the concept of maintenance by experience because the onset of locomotion in infancy is associated with more flexible use of the two strategies during spatial search and coding tasks. It would be interesting to see whether older adults with mobility impairments, or who were more sedentary, would have more difficulty switching to an allocentric strategy than those without an impairment or those who were more physically active.

In summary, the concept of maintenance by experience not only highlights the enduring effects of locomotor experience, but offers an alternative way to conceptualize the relation between gait dysfunction and cognitive decline in the elderly. Rather than view the relation as unidirectional, i.e., neural and vascular changes lead to a deterioration in gait and cognitive function, with the deterioration in gait continuing as executive function becomes increasingly compromised, it may be more appropriate to view the relation as bidirectional. Impaired mobility is very likely to exacerbate cognitive impairment because it limits the interaction with the environment that is known to drive structural and functional changes in the brain. We will elaborate on this idea in the next section.

## What implications do motor disabilities have for psychological development?

We have already noted that infants who are delayed in the onset of locomotion for neurological or orthopaedic reasons have also been shown to be delayed in the development of spatial-cognitive skills. These findings have been confirmed in a recent longitudinal study of seven infants with spina bifida who were tested on three spatial-cognitive paradigms prior to and after the onset of independent crawling (Rivera, 2012). The first paradigm assessed visual proprioception in the moving room. The second paradigm assessed the ability to follow the point and gaze gesture of an experimenter and the third paradigm assessed the ability to extract the invariant form of an object that was presented in multiple sizes, orientations, and colors. Consistent with the Campos et al. (2009) findings, the infants showed marked improvements on each of the spatial-cognitive paradigms following the acquisition of crawling, which occurred at an average age of 19.6 months, well after typically-developing infants begin to crawl. In addition, we have also noted already that infants who engage in effortful forms of locomotion, like belly crawling, don't appear to profit, in terms of psychological consequences, from their locomotor experience. We suspect that at least some of the cognitive deficits that have been noted in older children and adults with motor disabilities might be attributable to a lack of locomotor experience or delays in locomotor experience, particularly if those delays straddle sensitive periods in the development of the psychological skills in question.

The idea that motoric limitations might contribute to limitations in perceptual and spatial-cognitive functioning in children with motoric disabilities is not new (e.g., Abercrombie, 1964, 1968; Kershner, 1974). Limited evidence currently exists, however, to support the idea and the current model in developmental pediatrics has a strong bias against motoric factors playing a role in the psychological development of children with disabilities (Anderson et al., 2013). A major problem with accepting a role for motoric factors in the psychological development of children with physical disabilities has been the difficulty associated with separating the role of brain damage from that of mobility impairment in any psychological deficits that are discovered. Brain damage is often the cause of the primary motor impairments seen in children with physical disabilities and that same damage is obviously implicated in any co-occurring spatial-cognitive deficits.

Despite the above-mentioned difficulties, there is clear evidence that limited opportunities to explore the environment can impede the development of spatial-cognitive skills. Notably, in reference to the previous section, navigation is one of the skills that is most severely affected. One of the first studies to examine the effects of limited exploration on the development of navigation skills was conducted by Simms (1987). We have already discussed the more flexible use of egocentric and allocentric spatial coding strategies that accompanies the shift to independent locomotion in typically developing children as well as the difficulties that older adults often have using allocentric strategies. The development of spatial coding does not end, however, once the child has acquired the ability to use allocentric strategies. Rather, it continues to develop as children learn routes to target locations and ultimately learn to integrate routes and landmarks into an overall representation of the environment (Piaget and Inhelder, 1948; Siegel and White, 1975). In Simms's (1987) study, nine young adults with spina bifida and nine able-bodied controls had to learn routes while being driven through a traffic-free road system and a busy village. Compared to able-bodied controls, the young people with spina bifida took significantly longer to learn a route, noticed fewer landmarks, were less able to mark routes on a map, and produced poorer hand drawn maps. Importantly, the participants' level of mobility was linked to spatial skill, with walkers performing better than wheelchair users.

More recent studies have confirmed that children with physical disabilities have difficulties acquiring spatial knowledge related to navigation (e.g., Foreman et al., 1989, 1990; Stanton et al., 2002; Wiedenbauer and Jansen-Osmann, 2006) and have demonstrated that the severity of motor disability and the severity of brain damage make independent contributions to spatial-cognitive impairments (Pavlova et al., 2007). The study by Foreman et al. (1990) is particularly revealing because it shows that active decision making may be one of the key mediators in the link between locomotion and the acquisition of spatial knowledge. In two experiments, 4–6 year-old children were tested for their ability to retrieve objects that were strategically positioned within a large room. The children were first familiarized with the object positions in one of four locomotor conditions: (1) independently walking between positions, (2) walking but being led by an experimenter, (3) passively transported in a wheelchair, or (4) passively transported in a wheelchair while directing the experimenter where to go. The results showed that children who walked independently or directed the experimenter while being pushed in the wheelchair performed most successfully on the task. Thus, control over decision making was the crucial determinant of spatial search performance following navigation through the room and not the means by which locomotion was achieved. This finding is important because it further highlights the distinction between the experiences that are associated with locomotion and the means by which locomotion is achieved. A considerable body of research with typically developing children now shows that *active* locomotion facilitates spatial search performance (Yan et al., 1998).

When the studies linking crawling experience with spatial-cognitive development in infants with spina bifida are combined with the studies showing spatial-navigational deficits in older children with physical disabilities, the evidence in favor of the hypothesis that impaired mobility contributes to impaired psychological development is already quite strong and growing stronger. Nevertheless, considerably more work needs to be done in this area before clinicians will accept the hypothesis without reservation. In the meantime, it is encouraging that some researchers and clinicians are already exploring the psychosocial benefits that might stem from early powered-mobility training in children with mobility impairments (e.g., Lynch et al., 2009; Ragonesi et al., 2010). Continued work in this broad area is imperative given the millions of children with physical disabilities world-wide who could potentially profit from our deeper understanding of the relation between locomotor impairments and psychological deficits.

## Concluding comments

The onset of independent locomotion is a momentous event in human development. It marks a major transition toward independence from caregivers, it creates an explosion of new choices for the infant, and it heralds a remarkably broad set of changes in psychological functioning. Overwhelming evidence suggests that locomotion is not merely a maturational antecedent to these changes. Rather, the changes are a function of the specific experiences that accompany moving oneself through the world. Consistent with the idea that development is probabilistic, infants could potentially be exposed to these experiences in non-locomotor ways and thus acquire the psychological skills through alternative developmental pathways. However, the acquisition of these skills through alternative pathways in the typically-developing infant is likely rare. What makes locomotion significant is that it virtually guarantees that infants will encounter the requisite experiences that drive a host of important psychological changes; many of which were not documented in this paper and many of which remain to be discovered. Even though self-produced locomotion may not be necessary for these changes to take place, locomotion is significant because in the ecology of the typically-developing infant it is the most common means by which these changes happen.

The enduring significance of locomotion stems from the fact that, once acquired, it is typically maintained; though it also becomes more effectively controlled, more efficient, and more adaptable to a range of different morphological and contextual constraints. Locomotion can thus serve as a permanent framework for the maintenance of the psychological skills it helped to engender in the first place. Moreover, the onset of new locomotor skills, like walking or running, will likely have consequences for the development of more sophisticated psychological skills. This hypothesis is already being tested. The maintenance idea has important implications for our understanding of the declines in psychological functioning that occur when locomotion is compromised by aging, injury, disease, or disability, and it deserves to be scrutinized much more carefully. Equally worthy of further scrutiny are the psychological consequences associated with motor disabilities that delay the acquisition of independent locomotion or impair its quality once acquired. Many questions remain unanswered about the specific processes by which locomotion brings about psychological changes as well as the specific changes in neural structure and function that can be tied to locomotion. Questions also remain about the acquisition of other motor skills that may have implications for psychological development. Addressing all of these questions could markedly enhance not only our understanding of the specific role that locomotion plays in psychological processes across the lifespan, but also the broader role that action plays in those same processes. Ultimately, we argue that the acquisition of any skill that dramatically changes the relation between the person and the environment must have consequences for psychological functioning. This idea has significant implications for the way we view and understand human development.

### Conflict of interest statement

The authors declare that the research was conducted in the absence of any commercial or financial relationships that could be construed as a potential conflict of interest.
